# Durable Response to Brentuximab Vedotin Plus Cyclophosphamide, Doxorubicin, and Prednisone (BV-CHP) in a Patient with CD30-Positive PTCL Arising as a Post-Transplant Lymphoproliferative Disorder (PTLD)

**DOI:** 10.3390/curroncol28060426

**Published:** 2021-12-02

**Authors:** Jennifer Hong, William T. Johnson, Saritha Kartan, Anitha S. Gonsalves, Jonathan M. Fenkel, Jerald Z. Gong, Pierluigi Porcu

**Affiliations:** 1 Department of Hematology and Oncology, Lankenau Medical Center, Wynnewood, PA 19096, USA; 2 Department of Medical Oncology, Memorial Sloan Kettering Cancer Center, New York, NY 10065, USA; JohnsoW3@mskcc.org; 3 Department of Medical Oncology, Thomas Jefferson University Hospital, Philadelphia, PA 19107, USA; Saritha.Kartan@jefferson.edu (S.K.); Pierluigi.Porcu@jefferson.edu (P.P.); 4 Department of Radiology, Pottstown Hospital, Pottstown, PA 19464, USA; anvids@gmail.com; 5 Department of Gastroenterology, Thomas Jefferson University Hospital, Philadelphia, PA 19107, USA; Jonathan.Fenkel@jefferson.edu; 6 Department of Pathology, Thomas Jefferson University Hospital, Philadelphia, PA 19107, USA; Jerald.Gong@jefferson.edu

**Keywords:** post-transplant lymphoproliferative disorders, T-cell, peripheral T-cell lymphoma, NOS, CD30, brentuximab, BV-CHP

## Abstract

T-cell PTLDs are lymphoid proliferations that develop in recipients of SOT or allogeneic HSCT. They carry an extremely poor prognosis with a reported median survival of only 6 months. The infrequency with which they are encountered makes treatment a challenge due to the lack of prospective trials to guide management. The significantly higher risk of morbidity and mortality in T-cell PTLD, compared to B-cell PTLD, underscores the challenge of treating these patients and the need for new therapeutic options. Brentuximab vedotin, an ADC targeting CD30, is FDA-approved in combination with CHP as front-line treatment for patients with CD30 expressing PTCL. Herein we report a case of CD30-positive T-cell PTLD that was successfully treated with BV-CHP, suggesting the added value of the addition of BV to chemotherapy, contributing to our patient’s long and ongoing progression-free survival. To our knowledge, this is the first documented case of successful treatment using BV-CHP for a CD30-positive, EBV-negative, late T-cell PTLD.

## 1. Introduction

Post-transplant lymphoproliferative disorders (PTLD) are a rare but serious complication following solid organ (SOT) and allogeneic hematopoietic stem-cell transplantation (HSCT). The majority of PTLDs arise from B-cells and are frequently associated with EBV reactivation, especially when they occur early post-transplant (<12 months). PTLD cases arising from T-cells and NK cells are much less common (2–15% of all PTLD) and more heterogeneous than B-cell PTLD. They often develop after a long latency (median 9 years post-transplant), and are less frequently associated with EBV [1,2,3,4]. Natural history and treatment outcome data for T/NK-cell PTLD are limited, with less than 200 cases reported in the literature [1,2,5]. Patients often have aggressive disease and a poor prognosis (median survival ~6 months) even when treated with aggressive multi-agent chemotherapy regimens, such as cyclophosphamide, doxorubicin, vincristine, and prednisone (CHOP) [2,6].

Brentuximab vedotin (BV) is an antibody-drug conjugate (ADC) targeting the surface activation marker CD30. In a Phase II study in 34 evaluable patients, BV was effective in the treatment of relapsed and refractory, non-anaplastic, CD30-positive T-cell lymphomas, with an overall response rate (ORR) of 41% [7]. Based on a large, randomized Phase III trial (ECHELON-2) this showed the superior progression-free survival (PFS), and overall survival (OS) compared to standard of care CHOP. The combination of BV with doxorubicin-based chemotherapy (BV-CHP) is now FDA approved for the front-line treatment of CD30-expressing peripheral T-cell lymphomas (PTCL) [8,9]. While approximately 70% of the 452 patients enrolled in ECHELON-2 had systemic anaplastic large-cell lymphoma (sALCL), the FDA label for BV-CHP in the U.S. includes other types of CD30-expressing PTCL. This introduced BV-CHP as an additional option for the front-line management of patients with CD30-expressing PTCLs. Despite the fact that the trial was not able to detect outcome differences in non-ALCL PTCL, BV-CHP is now generally preferred to CHOP in these patients and is included in NCCN guidelines (category 2A), based on a toxicity profile that is overall comparable to CHOP (with the exception of sensory neuropathy) and the hope that the targeting of CD30 will produce additional clinical benefit [8].

There are anecdotal responses to single-agent BV in patients with T-cell PTLD, suggesting that BV can be effective in CD30-expressing T-cell lymphomas arising in immunocompromised patients [6,8]. While ECHELON-2 excluded immunocompromised patients, considering the very poor survival outcomes reported with CHOP chemotherapy in T-cell PTLD, there is pharmacological justification and clinical motivation for the use of BV-CHP in these patients. Here, we report a case of CD30-positive T-cell PTLD with a very prolonged response to BV-CHP.

## 2. Case

A 72-year-old white male with end-stage liver disease due to cryptogenic cirrhosis underwent a deceased-donor orthotopic liver transplantation (OLT) in 2010. The post-transplant immunosuppression (IS) regimen included tacrolimus 5 mg twice daily, prednisone 20 mg daily, and mycophenolate mofetil (MMF) 1000 mg twice daily.

He remained relatively well until seven years post-transplant when he presented with right foot pain and right lower extremity swelling for a duration of 4 weeks. Doppler ultrasound showed an acute right popliteal, tibial, and peroneal deep venous thrombosis for which he was prescribed apixaban. Two months after the initiation of anticoagulation therapy, he presented with a recurrence of right lower extremity swelling, increased pain and numbness, and a new weakening of the right foot. Further history revealed progressive fatigue, dyspnea, and a 30-pound weight loss over 3 months.

**Figure 1:**
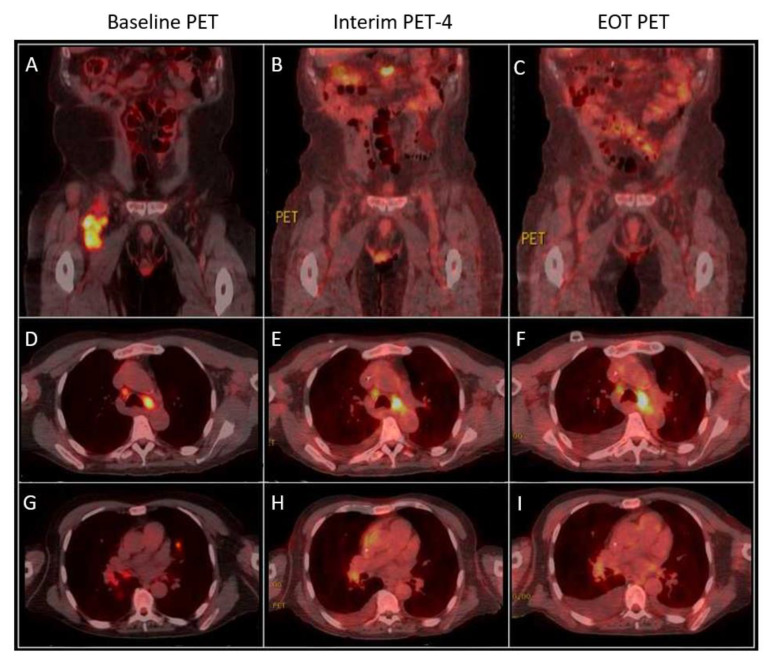
Comparison of baseline, interim, and end-of-treatment (EOT) PET-CT. (**A**) An abnormal 5.8 × 3.9 cm^2^ soft tissue mass (SUV 11.9) adjacent to the proximal right femur demonstrates near complete resolution after cycle 4 (SUV 1.68) (**B**) and complete resolution after cycle 6 (**C**). (**D**) Enhancing mediastinal adenopathy (max SUV 13.4, Deauville Score 5) with significant improvement (SUV 9.3, Deauville Score 4) after cycle 4 (**E**) maintained after cycle 6 (Deauville Score 4) (**F**). (**G**) Abnormal FDG uptake of numerous subcentimeter bilateral pulmonary nodules (max SUV 8.4) with complete resolution after cycle 4 (**H**) and maintained after cycle 6 (**I**).

A computed tomography (CT) of the chest showed scattered lung nodules with multiple areas of bilateral thoracic lymphadenopathy. A positron emission tomography (PET-CT) revealed the abnormal FDG uptake of numerous, sub-centimeter bilateral pulmonary nodules (max SUV 8.4) with mediastinal (1.6 cm, max SUV 13.4), bilateral hilar, right femoral, inguinal adenopathy, and abnormal FDG uptake in a soft tissue mass adjacent to the proximal right femur (5.8 × 3.9 cm^2^, max SUV 11.9) (Figure 1).

**Figure 2:**
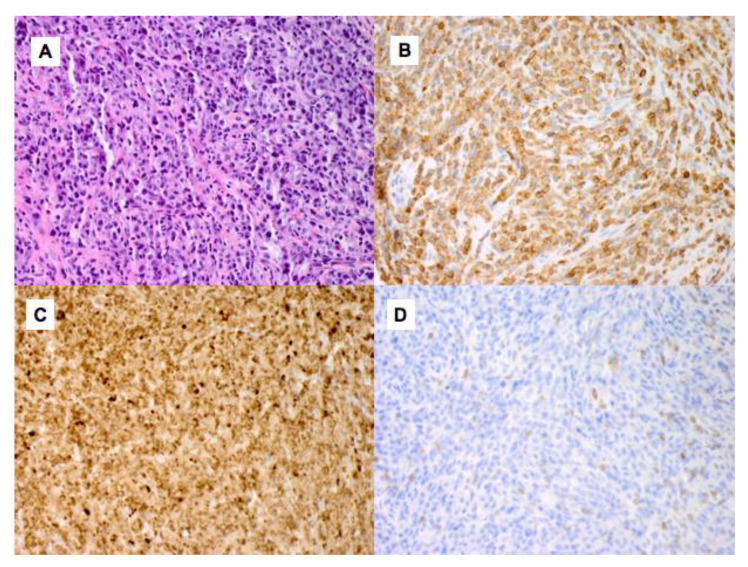
Histologic morphology and immunohistochemical stains on the soft tissue mass. (**A**) The tumor shows a diffuse infiltration of large, atypical lymphocytes in a fibrohistiocytic background (Hematoxylin and Eosin ×400). The lymphoma cells express CD3 (**B**), CD30 (30% of lymphocytes) (**C**) and are negative for CD5 (×400) (**D**).

An excisional biopsy of the soft tissue mass showed diffuse infiltration with atypical monomorphic lymphoid cells with large regions of necrosis (Figure 2). By immunohistochemistry the tumor cells expressed CD3, CD4, CD30 (30%), and BCL-2, and were negative for CD5, CD8, CD10, CD20, CD21, TIA-1, perforin, T-cell receptor (TCR) gamma, and ALK-1. In situ hybridization for Epstein–Barr virus (EBV)-encoded RNA was negative and plasma EBV DNA was not detectable. The proliferation index estimated by Ki-67 staining was 70–80%. TCR gene rearrangement analysis showed a monoclonal band for both the TCR gamma and beta genes. A bone marrow biopsy showed normocellular bone marrow, with trilineage hematopoietic maturation, and no lymphoid aggregates or atypical lymphoid cells, but with a mononoclonal TCR rearrangement identical to that found in the soft tissue mass, consistent with a low-level involvement by T-cell lymphoma. He was diagnosed with stage IV CD30-positive T-cell PTLD, peripheral T-cell lymphoma (PTCL), which was not otherwise specified (NOS).

At the time of the PTLD diagnosis, IS therapy consisted only of tacrolimus 1 mg twice daily. After discussion with the transplant team, the tacrolimus dose was decreased to 0.5 mg twice daily, and CHOP-21 chemotherapy was initiated. However, based on the emerging ECHELON-2 outcomes with the addition of brentuximab vedotin (BV) to an anthracycline-based chemotherapy backbone in CD30-positive PTCL, after two cycles of CHOP, the patient’s chemotherapy regimen was changed to BV-CHP [10,11,12]. BV was dosed at 1.8 mg/kg with CHP every 3 weeks with G-CSF support, according to the published ECHELON-2 regimen [9,10]. Cycle 3 (BV-CHP #1) was complicated by neutropenic sepsis, and BV was reduced to 1.3 mg/kg for cycles 4–6. Cycle 4 (BV-CHP #2) was again complicated by neutropenic sepsis, and cyclophosphamide and doxorubicin dosing were reduced by 25% for the remaining cycles 5 and 6. Interim PET-CT after cycle 4 showed a complete resolution of previously seen pulmonary metastases, a decrease in the mediastinal (1.3 cm, max SUV 9.3; from pre-treatment 1.6 cm, max SUV 13.4) and hilar adenopathy, and a near complete resolution of the soft tissue mass in the right anterior thigh (2.4 × 2.3 cm^2^, max SUV 1.68, from pre-treatment 5.8 × 3.9 cm^2^, max SUV 11.9), overall consistent with a partial response (PR). End-of-treatment PET-CT after cycle 6 showed stable disease in the mediastinal (1.3 cm, max SUV 10.25) and hilar adenopathy, and no new or recurrent adenopathy, with complete resolution of the previously seen right lower extremity soft tissue mass, consistent with ongoing PR (Figure 1). Maintenance therapy with single-agent BV was discussed and started, but after one BV maintenance dose he was again admitted with a neutropenic febrile episode and the patient requested to stop all therapy, as he was satisfied with the achieved response.

Following completion of front-line therapy, his course was complicated by elevations of alkaline phosphatase (peak 417 U/L 3 months post-treatment initiation, from pre-treatment 97 U/L) and AST (peak 75 U/L 11 months post-treatment initiation, from pre-treatment 33 U/L) levels. ALT remained within normal limits. A liver biopsy completed 8 months after treatment initiation showed minimal lobular inflammation, but no evidence of portal inflammation, bile duct injury, or endothelitis, as seen with acute cellular rejection, and no ductopenia, as seen with chronic rejection.

Due to chemotherapy-induced thrombocytopenia and an episode of duodenal ulcer bleeding, his anticoagulation was briefly interrupted and then re-initiated after endoscopy clipping and the recovery of thrombocytopenia. His known DVTs were monitored with serial Doppler ultrasound, which demonstrated a chronically dilated, thrombosed right popliteal vein with post-thrombotic venous changes, representing chronic thrombosis despite being on anticoagulation. No additional complications were observed. Neutropenia and thrombocytopenia normalized 4 months after the last dose of BV, with mild persistent anemia (8–11 g/dL). He remains alive and well 25 months since his diagnosis without clinical or radiographic evidence of progression.

## 3. Discussion

PTLDs are lymphoid proliferations that develop in recipients of SOT or allogeneic HSCT [13,14]. The risk of PTLD is highest in recipients of multivisceral and intestinal transplants (<20%), followed by lung (3–10%), heart (2–8%), liver (1–5.5%), pancreas (0.5–5%), and kidney (0.8–2.5%) [6]. Additionally, the variable intensity of induction immunosuppression and the cumulative doses of maintenance immunosuppressive therapy that warranted in these transplants played a role in the development of PTLD, although the individual effect of each immunosuppressive agent is not clear [15,16].

Monomorphic PTLDs, the most common type, are classified based on morphologic and immunophenotypic criteria according to the WHO-defined lymphoma entity that they most closely resemble [13,17]. The majority of monomorphic PTLD arise from B-cells, whereas cases arising from T-cell PTLD are less common and more heterogeneous, with PTCL-NOS being the most common histology, as seen in this patient [2,4,13,14,15].

Monomorphic T-cell PTLDs carry an extremely poor prognosis. Owing to their rarity, late occurrence and EBV-negative status, much of the knowledge guiding the management of T-cell PTLD is derived from findings in single case reports and very small series. As such, these rare subtypes of T-cell PTLD are treated with subtype-specific treatment regimens, with either a combination or sequences of reduction in immunosuppression or multi-agent chemotherapy. As with our patient, patients diagnosed with T-cell PTLD, PTCL-NOS are treated with CHOP or CHOP-like regimens, similar to de novo nodal PTCL-NOS in immunocompetent patients, with very poor outcomes. The significantly higher risk of morbidity and mortality in T-cell PTLD, compared to B-cell PTLD, underscores the challenge of treating these patients and the need for new therapeutic options.

CD30 is a membrane-bound glycoprotein belonging to the tumor-necrosis-factor receptor superfamily (TNFRSF) whose expression in healthy tissues is limited to activated lymphocytes and is upregulated on malignant cells, most notably in Hodgkin’s lymphoma and anaplastic large-cell lymphoma [6,12]. The frequency of CD30 expression in T-cell PTLD is not well known, but positive staining was observed in 33% of cases in one series and in up to 60% of PTCL, NOS in immunocompetent patients [5,12,18].

In 2018, the FDA approved BV in combination with chemotherapy as front-line treatment for patients with CD30-expressing PTCL, based on the results of the pivotal ECHELON-2 trial. BV-CHP was superior to CHOP for the study’s primary endpoint, PFS (48.2 months vs. 20.8 months, HR 0.71), and for all secondary endpoints, including CR rate (68% vs. 56%), ORR (83% vs. 72%), and OS (HR 0.66; median OS was not reached in either arm). BV-CHP resulted in a 29% and 34% reduction in the risk of disease progression and death, respectively [9]. Patients were designated as CD30-positive if ≥10% of neoplastic cells expressed CD30.

The incidence and severity of treatment-related adverse events was similar in the BV-CHP and CHOP treatment groups in ECHELON-2. While the ECHELON-2 trial did not include immunocompromised patients, neutropenia and febrile neutropenia were common in both the BV-CHP and CHOP cohort (38% in both and 18% vs. 15%, respectively). The most common toxicity was peripheral neuropathy (45% vs. 41%, respectively). Other common toxicities included Grade >3 infections (19% vs. 14%) and anemia (21% vs. 16%). Treatment discontinuation and death as a result of adverse events were also similar between the BV-CHP and CHOP groups, reported as 6% vs. 7% and 3% vs. 4%, respectively. Other adverse events of any grade in order of occurrence included nausea, diarrhea, constipation, alopecia, vomiting, and fatigue [9].

The spectrum of toxicities experienced by our patient is consistent with that observed in the ECHELON-2 population, with the exception of their severity and high recurrence rates, in particular for febrile neutropenia, likely due to his immunocompromised status as a result of his post-transplant status and immunosuppressant use. Considering the high risk of progression with a PR, we attempted maintenance therapy with single-agent BV after BV-CHP but recurrent toxicities and patient preference led to discontinuation of therapy.

## 4. Conclusions

The expression of CD30 (30%) in this patient’s T-cell PTLD provided the opportunity to add BV to standard front-line cytotoxic chemotherapy with CHOP. The historically poor outcomes with CHOP in T-cell PTLD suggest that the addition of BV to chemotherapy after an initial course of 2 cycles of CHOP and reduction in immunosuppression may have contributed to his remarkably long and ongoing progression-free survival 25 months since his diagnosis. Adding BV to front-line chemotherapy was challenging but possible in this patient. To our knowledge, this is the first documented case of successful treatment using BV-CHP for a CD30-positive, EBV-negative, late T-cell PTLD.

## Data Availability

No new data were created or analyzed in this study. Data sharing is not applicable to this article.

## References

[1] Caillard S., Porcher R., Provot F., Dantal J., Choquet S., Durrbach A., Morelon E., Moal V., Janbon B., Alamartine E. (2013). Post-transplantation lymphoproliferative disorder after kidney transplantation: Report of a nationwide French registry and the development of a new prognostic score. Am. J. Clin. Oncol..

[2] Swerdlow S.H. (2007). T-cell and NK-cell posttransplantation lymphoproliferative disorders. Am. J. Clin. Pathol..

[3] Draoua H.Y., Tsao L., Mancini D.M., Addonizio L.J., Bhagat G., Alobeid B. (2004). T-cell post-transplantation lymphoproliferative disorders after cardiac transplantation: A single institutional experience. Br. J. Haematol..

[4] Montanari F., Bhagat G., Clark-Garvey S., Seshan V., Zain J., Diefenbach C., Mccormick E., Crook M., Conroy M., O’connor O.A. (2010). Monomorphic T-cell post-transplant lymphoproliferative disorders exhibit markedly inferior outcomes compared to monomorphic B-cell post-transplant lymphoproliferative disorders. Leuk. Lymphoma.

[5] Margolskee E., Jobanputra V., Jain P., Chen J., Ganapathi K., Nahum O., Levy B., Morscio J., Murty V., Tousseyn T. (2016). Genetic landscape of T- and NK-cell post-transplant lymphoproliferative disorders. Oncotarget.

[6] Herreman A., Dierickx D., Morscio J., Camps J., Bittoun E., Verhoef G., De Wolf-Peeters C., Sagaert X., Tousseyn T. (2013). Clinicopathological characteristics of posttransplant lymphoproliferative disorders of T-cell origin: Single-center series of nine cases and meta-analysis of 147 reported cases. Leuk. Lymphoma.

[7] Horwitz S.M., Advani R.H., Bartlett N.L., Jacobsen E.D., Sharman J.P., O’Connor O.A., Siddiqi T., Kennedy D.A., Oki Y. (2014). Objective responses in relapsed T-cell lymphomas with single-agent brentuximab vedotin. Blood.

[8] Barta S.K., Gong J.Z., Porcu P. (2019). Brentuximab vedotin in the treatment of CD30+ PTCL. Blood.

[9] Horwitz S., O’Connor O.A., Pro B., Illidge T., Fanale M., Advani R., Bartlett N.L., Christensen J.H., Morschhauser F., Domingo-Domenech E. (2019). Brentuximab vedotin with chemotherapy for CD30-positive peripheral T-cell lymphoma (ECHELON-2): A global, double-blind, randomised, phase 3 trial. Lancet.

[10] Fanale M.A., Horwitz S.M., Forero-Torres A., Bartlett N.L., Advani R.H., Pro B., Chen R.W., Davies A., Illidge T., Huebner D. (2014). Brentuximab vedotin in the front-line treatment of patients with CD30+ peripheral T-cell lymphomas: Results of a phase I study. Am. J. Clin. Oncol..

[11] Fanale M.A., Horwitz S.M., Forero-Torres A., Bartlett N.L., Advani R.H., Pro B., Chen R.W., Davies A., Illidge T., Uttarwar M. (2018). Five-year outcomes for frontline brentuximab vedotin with CHP for CD30-expressing peripheral T-cell lymphomas. Blood.

[12] Van Der Weyden C.A., Pileri S.A., Feldman A.L., Whisstock J., Prince H.M. (2017). Understanding CD30 biology and therapeutic targeting: A historical perspective providing insight into future directions. Blood Cancer J..

[13] Dierickx D., Tousseyn T., Gheysens O. (2015). How I treat posttransplant lymphoproliferative disorders. Blood.

[14] Swerdlow S.H., Campo E., Pileri S.A., Harris N.L., Stein H., Siebert R., Advani R., Ghielmini M., Salles G.A., Zelenetz A.D. (2016). The 2016 revision of the World Health Organization classification of lymphoid neoplasms. Blood.

[15] Al-Mansour Z., Nelson B.P., Evens A.M. (2013). Post-transplant lymphoproliferative disease (PTLD): Risk factors, diagnosis, and current treatment strategies. Curr. Hematol. Malig. Rep..

[16] Dierickx D., Habermann T.M. (2018). Post-transplantation lymphoproliferative disorders in adults. N. Engl. J. Med..

[17] Sabattini E., Bacci F., Sagramoso C., Pileri S.A. (2010). WHO classification of tumours of haematopoietic and lymphoid tissues in 2008: An overview. Pathologica.

[18] Bossard C., Dobay M.P., Parrens M., Lamant L., Missiaglia E., Haioun C., Martin A., Fabiani B., Delarue R., Tournilhac O. (2014). Immunohistochemistry as a valuable tool to assess CD30 expression in peripheral T-cell lymphomas: High correlation with mRNA levels. Blood.

